# Gaussian primitives for deformable image registration

**DOI:** 10.1016/j.phro.2025.100821

**Published:** 2025-08-08

**Authors:** Jihe Li, Xiang Liu, Fabian Zhang, Xixin Cao, Joachim M. Buhmann, Ye Zhang, Xia Li

**Affiliations:** a School of Software and Microelectronics, Peking University, Beijing, 100871, China; b School of Computing, National University of Singapore, Singapore, 119077, Singapore; c Department of Computer Science, ETH Zürich, Zürich, 8092, Switzerland; d Center for Proton Therapy, Paul Scherrer Institut, Villigen, 5232, Switzerland

**Keywords:** Deformable image registration, Gaussian primitives, Motion representation

## Abstract

**Background and Purpose:**

Deformable image registration (DIR) plays a critical role in radiotherapy by compensating for anatomical deformations. However, existing iterative and data-driven methods are often hindered by computational inefficiency or limited generalization. In response, our objective was to develop a novel optimization-based DIR method that reduces computational overhead and preserves the robust generalization of iterative methods while enhancing interpretability.

**Materials and Methods:**

We proposed GaussianDIR, a novel DIR framework that explicitly represents the deformation field using a sparse set of adaptive Gaussian primitives. Each primitive is characterized by its centre, covariance, and associated local rigid deformation. Voxel-wise displacements are derived via blending the local rigid deformations of neighbouring primitives, enabling flexible yet efficient motion modelling.

**Results:**

On DIRLab lung dataset, GaussianDIR achieved a target registration error (TRE) of 1.00±1.11 millimeters in about 2.5 s, offering an effective trade-off between speed and precision for high-resolution images. On OASIS brain and ACDC cardiac datasets, the Dice similarity coefficient (DSC) improved from 80.6% to 81.3% and from 81.0% to 81.2% over previous state-of-the-art methods, respectively. Moreover, we compared the generalization performance of GaussianDIR and a data-driven method on IXI dataset, and found that GaussianDIR outperformed the data-driven method by 6.3% in DSC.

**Conclusion:**

GaussianDIR combines high registration accuracy with computational efficiency, interpretability, and strong generalization performance. It challenged the conventional notion that iterative methods were inherently slow and overcomed the generalization limitations of data-driven methods, with potential for real-time clinical applications in radiotherapy.

## Highlights

• A novel explicit representation for deformable image registration is proposed.
• Equip case-specific optimization with high efficiency.
• Enables clear interpretability through explicit Gaussian primitives.
• Achieves 1.00±1.11 millimeter landmark error in about 2.5 s on DIRLab dataset.
• Improves Dice score from 80.6% to 81.3% on the OASIS brain dataset.

## 1 Introduction

Deformable image registration (DIR) has become an essential component of modern radiotherapy, addressing anatomical variations and enhancing precision throughout the fractionated treatment workflow. Given a pair of images (fixed and moving), DIR aims to compute a displacement vector field (DVF) that warps the moving image to align with the fixed image. Unlike rigid or affine registration, DIR can handle complex, non-linear deformations, making it indispensable for applications in radiotherapy such as contour propagation [1], [2], dose accumulation [3], [4], and motion modelling [5], [6].

DIR methods can be broadly categorized into classical iterative optimization-based, deep learning (DL)-based, and implicit neural representations (INR)-based approaches. Classical iterative methods [7], [8], [9], [10], [11] formulate DIR as a variational optimization problem that relies heavily on mathematical priors. These methods are robust and generalize well across various imaging modalities and anatomical regions, while they often suffer from high computational costs. For instance, LDDMM [8] requires solving complex partial differential equations, which results in significant computational overhead, especially when dealing with images of large size [12].

The rise of DL has introduced data-driven approaches that leverage large datasets to train neural networks to predict dense DVF from pairs of fixed and moving images [13], [14], [15], [16], [17]. One benefit of DL-based methods is incorporating weak supervision from anatomical landmarks or segmentation maps [16], [18] during training. However, DL-based methods are limited by their dependency on large and diverse datasets, which are often complex and expensive to obtain in medical settings. Moreover, they struggle with generalization when applied to data distributions different from the training set, significantly limiting their practical utility [18].

INR-based methods [19], [20], [21] use multi-layer perceptrons (MLPs) to represent the coordinates mapping function between fixed and moving images. One of their key designs is to perform mini-batch gradient descent to optimize the network, enabling relatively fast processing despite facing high-resolution DVF. However, INR-based methods often suffer from challenges in capturing sharp and complex deformations and lack interpretability due to their implicit nature, which still lacks real-time speed. Recently, 3D Gaussian splatting (3DGS) [22] has emerged as a more efficient and interpretable approach for 3D reconstruction, using mobile and elastic 3D Gaussian together with spherical harmonics (SH) to model the appearance of local regions. To improve rendering efficiency, it utilizes truncated Gaussian to perform α-blending.

Inspired by 3DGS, we innovatively explored the application of Gaussian primitives in DIR. We aimed to combine registration efficiency and generalization, which existing methods struggle to achieve while enhancing interpretability compared to INR-based and DL-based methods. Therefore, we introduced GaussianDIR, a novel case-specific optimization DIR approach beyond traditional grid-based [9], [23] or voxel-based representations [7], [24]. It models the DVF using a set of mobile and flexible 3D Gaussian primitives, each representing the deformation of a local region, as anatomical tissues within a localized area typically undergo similar deformations. This formulation offers three key advantages. First, it enables efficient representation and optimization of deformations while maintaining strong generalization due to its case-specific optimization paradigm. In contrast, DL-based methods following a training–inference paradigm suffer from limited generalization. Second, unlike traditional B-spline-based methods that use fixed grid-based control points, the dynamic nature of GaussianDIR provides greater flexibility. Third, employing an explicit representation of Gaussian primitives enhances interpretability, overcoming the limitations inherent in INR and DL models based on implicit neural networks. These strengths highlight its potential for clinical applications, particularly in time-sensitive scenarios.

## 2 Materials and methods

### 2.1 Problem statement

The task addressed in this paper is pairwise image registration, focusing on calculating the DVF, which represents the voxel-wise displacement required to align a moving image with a fixed image. Specifically, considering a pair of images, $I_{m}$ and $I_{f}$, with voxels $x\in Z^{3}$, DIR estimates the DVF ϕ, which maps each voxel of $I_{m}$ to its corresponding location in $I_{f}$. The optimization problem for DIR is formulated as follows:

(1) $$\overset{ˆ}{\phi }=\arg\underset{\phi }{\min}L_{s}\left(I_{m}\left(x+\phi \left(x\right)\right),I_{f}\left(x\right)\right)+R\left(\phi \right),$$

where $I_{m}$ and $I_{f}$ correspond to moving and fixed images, and $L_{s}$ measures the image similarity. The term R represents a regularization applied to the DVF ϕ to constrain the solution space, avoiding singular solutions.

**Fig. 1:**
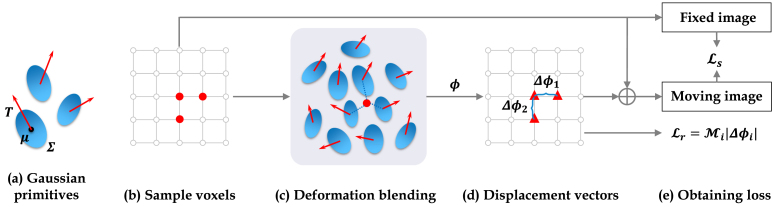
Pipeline of GaussianDIR: (a) Blue ellipses represent 3D Gaussians parameterized by centre position μ and covariance matrix Σ. Red arrows indicate the local deformations T. (b) Voxel groups are sampled from the volume to enable mini-batch optimization and total variation regularization. (c) Local deformations of neighbouring Gaussian primitives are blended, with neighbours identified using the KNN algorithm at two different scales. (d) Deformation vectors ϕ are obtained after deformation blending. (e) The similarity loss $L_{s}$ and regularization loss $L_{r}$ are then calculated. M represents the mean operation. (For interpretation of the references to colour in this figure legend, the reader is referred to the web version of this article.)

### 2.2 Gaussian representation and optimization

Fig. 1 provides a visual overview of the proposed approach, illustrating its sequential steps and key interactions. Each Gaussian primitive is employed to model local deformations. The displacement vector of each voxel is determined by blending the local rigid deformations of its neighbouring Gaussian primitives, similar to the truncated Gaussian approach in 3DGS. To accommodate varying deformation complexities and mitigate convergence to local minima, we introduced adaptive density control and multiscale primitives, elaborated in Supplementary Material A.1 and A.2, respectively. The parameters of the Gaussian primitives are optimized to maximize image similarity while preserving the smoothness of the DVF. In the following, we furnish a detailed explanation of Gaussian primitives, deformation blending and parameter optimization.

#### 2.2.1 Gaussian primitives

Our proposed approach models the deformation field using a collection of sparsely distributed Gaussian primitives, offering an explicit solution in contrast to the implicit counterpart used in IDIR [19]. As shown in Fig. 1(a), the geometric properties of each Gaussian primitive $G_{i}$ are defined by its centre position $\mu_{i}$ and covariance matrix $\Sigma_{i}$, which is decomposed as $\Sigma_{i}=Q_{i}S_{i}S_{i}^{T}Q_{i}^{T}$. Here, $S_{i}$ is a scaling matrix parameterized by a diagonal vector $s_{i}\in R^{3}$, and $Q_{i}\in \mathrm{SO}\left(3\right)$ is a rotation matrix represented by a quaternion $q_{i}\in R_{i}^{4}$. To describe the deformation, each Gaussian primitive is associated with a learnable local deformation $T_{i}\in \mathrm{SE}\left(3\right)$, which is further decomposed into a rotation matrix $R_{i}\in \mathrm{SO}\left(3\right)$, represented by a quaternion $r_{i}\in R^{4}$, and a translation vector $t_{i}\in R^{3}$. Consequently, the Gaussian primitives are parameterized as:

(2) $$G_{i}=\left\{\left(\mu_{i},s_{i},q_{i},r_{i},t_{i}\right)|i\leq N\right\},$$

where N is the number of Gaussian primitives.

#### 2.2.2 Deformation blending

The subsequent procedure is to calculate the dense deformation field for the volume. Inspired by α-blending [22], we employed the α-deformation-blending, which determines the displacement of a voxel based on its surrounding Gaussian primitives. Specifically, we performed a K-nearest neighbour (KNN) search to identify K closest Gaussian primitives of the voxel $x_{j}$. The blending weights $w_{jk}$ for voxel $x_{j}$ and Gaussian primitive $G_{k}$ are calculated as such:

(3) $$w_{jk}=\frac{{\overset{ˆ}{w}}_{jk}}{\underset{k\in N_{j}}{\sum }{\overset{ˆ}{w}}_{jk}},$$

(4) $${\overset{ˆ}{w}}_{jk}=\frac{1}{\sqrt{{\left(2\pi \right)}^{3}\left|\Sigma_{k}\right|}}\exp\left(-\frac{1}{2}{\left(x_{j}-\mu_{k}\right)}^{T}\Sigma_{k}^{-1}\left(x_{j}-\mu_{k}\right)\right),$$

where $N_{j}$ denotes the indices of the K nearest Gaussian primitives of voxel $x_{j}$.

Each Gaussian primitive impacts a local region in a 3D volume, applying the aforementioned SE(3) transformation to the covered voxels. The transformed position component of the voxel $x_{j}$, contributed by $G_{k}$, is denoted as ${\overset{ˆ}{x}}_{jk}$, which is determined by the following equation:

(5) $${\overset{ˆ}{x}}_{jk}=R_{k}\left(x_{j}-\mu_{k}\right)+\mu_{k}+t_{k}$$

Given the normalized blending weights and the transformed position components, we can calculate the displacement vector $\phi_{j}$ as the weighted sum of the local rigid deformation $T_{jk}$:

(6) $$\phi_{j}=\underset{k\in N_{j}}{\sum }w_{jk}T_{jk},\;T_{jk}={\overset{ˆ}{x}}_{jk}-x_{j}.$$

#### 2.2.3 Optimization

We employed the mini-batch gradient descent algorithm to optimize the parameters of Gaussian primitives, similar to the strategy used in IDIR [19]. Specifically, each mini-batch contains B=20,000 randomly sampled voxels. To guide the optimization process, we utilized negative normalized cross-correlation (NCC) [25] as the similarity loss $L_{s}$. Furthermore, we incorporate the total variation (TV) regularization [9], denoted as $L_{r}$, to enhance the smoothness of the DVF. However, traditional TV regularization is designed for whole images and is incompatible with our mini-batch-based optimization strategy. To address this, we developed an efficient mini-batch-based TV regularization method. Specifically, in each iteration, we randomly sampled $\frac{1}{D+1}B$ voxels, where D is the image dimension. For each sampled voxel, we also included its neighbouring voxels in three orthogonal directions to compute the TV loss. A 2D illustration of the TV regularization method is shown in Fig. 1(b). This sampling strategy reduces computational overhead while preserving the regularization effect. The final loss function is thus expressed as $L=L_{s}+\lambda L_{r}$, where λ is the weight balancing the regularization term. Detailed mathematical definitions of $L_{s}$ and $L_{r}$ are provided in Supplementary Material Section A.3.

### 2.3 Experimental settings

#### 2.3.1 Datasets

We conducted comprehensive experiments using multiple publicly available and anonymized datasets. Therefore, ethical approval was not required for this study. The datasets span lung computed tomography(CT), brain magnetic resonance imaging (MRI), and cardiac MRI images, specifically including:

##### DIR-Lab.

For lung CT evaluation, we adopted the DIR-Lab dataset [12], which consists of 10 lung 4D CT images, each annotated with 300 anatomical landmarks. We extracted the extreme inhalation and exhalation images as fixed and moving images, respectively, preserving the original resolution and unnormalized intensity values.

##### OASIS.

We utilized the Neurite version [14] of the OASIS dataset [26] for brain MRI evaluation, which includes 414 subjects with 35 anatomical labels. Following Jena et al. [18], we selected the last 50 subjects to form 49 test pairs, adhering to the pairing rules from the Learn2Reg challenge.

##### ACDC.

We used the ACDC dataset [27] to evaluate the performance of GaussianDIR on cardiac MRI registration, which contains 150 image pairs captured between the end-diastolic (ED) and end-systolic (ES) phases. The test set and preprocessing procedures in this work align with those used in CorrMLP [28].

##### IXI.

The IXI dataset,^2^ preprocessed by Transmorph [16], was employed to compare the generalization of our method with DL-based approaches. We followed the Transmorph protocol, using the provided test set and atlas-based registration strategy to assess registration accuracy.

#### 2.3.2 Metrics and comparison methods

We adopted standard evaluation metrics from prior works [9], [13], [19], [28], [29]: target registration error (TRE), Dice similarity coefficient (DSC), 95% maximum Hausdorff distance (HD95), and negative Jacobian determinant (NJD). TRE, DSC, and HD95 evaluate the alignment of anatomical landmarks or segmentation maps, while NJD quantifies the smoothness of the deformation fields. To evaluate its efficiency and accuracy, we conducted comparisons against a wide range of existing methods. These include classical approaches such as Demons [24], NiftyReg [23], Plastimatch [30], pTV [9], DIS-CO [31] and ConvexAdam [10], FireANTs [11], data-driven methods including VoxelMorph [13], SymNet [32], LapIRN [33], MJCNN [34], VIRNet [35], CorrMLP [28], and INR-based methods like NODEO [36], IDIR [19], ccIDIR [20]. Furthermore, to assess generalization capability, we compared the performance of GaussianDIR and TransMorph [16] on the IXI dataset. GaussianDIR employed the same set of hyperparameters tuned on the OASIS dataset, while TransMorph used a model trained on OASIS with weak anatomical map supervision.

#### 2.3.3 Implementation details

We initialized the positions of the Gaussian primitives at nodes of a 3D grid within PyTorch’s canonical space ${\left[-1,1\right]}^{3}$, while the local rigid deformations are set to the identity transformation (initial $t_{i}$s as 0). The Adam optimizer was used for 2000 iterations, with an initial warm-up phase for the learning rate, followed by a decay using a cosine annealing schedule. The weight λ for the regularization term was empirically set to 8. All experiments were run on an Nvidia RTX 4090 GPU.

## 3 Results

### 3.1 Performance comparisons

**Fig. 2:**
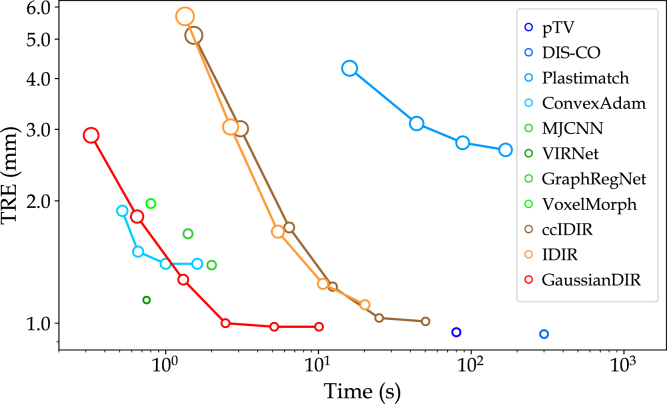
This line plot illustrated landmark error on the DIRLab dataset averaged across 10 cases with standard deviation represented by the size of the circles. Notably, GaussianDIR achieved the best trade-off between speed and accuracy.

**Fig. 3:**
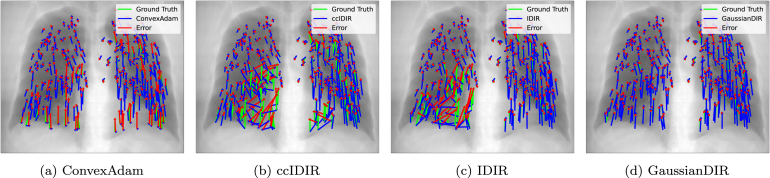
Landmark motion visualization for Case 8 of the DIRLab dataset. The visualization compared four methods, each trained for approximately 2.5 s. Blue, green and red arrows represent predicted motion, ground-truth motion and error, respectively. (For interpretation of the references to colour in this figure legend, the reader is referred to the web version of this article.)

Fig. 2 demonstrated that GaussianDIR offered an optimal trade-off between accuracy and computational efficiency. Although some DL-based methods, like VoxelMorph and MJCNN, achieved faster runtimes (under 1 s), their accuracy remained inferior to that of GaussianDIR. GaussianDIR could achieve a TRE of 1.00±1.11 millimeters (mm) in approximately 2.5 s, with only marginal runtime overhead relative to DL-based approaches. Classical optimization-based methods such as pTV and DIS-CO could achieve comparable or slightly higher accuracy, but at the cost of significantly increased runtime. Fig. 3 visualized landmark motion error comparisons after optimizing for about 2.5 s on Case 8 in the DIRLab dataset, clearly indicating that GaussianDIR yielded the most accurate alignment between the warped and fixed landmarks. Table 1 presents quantitative results and statistical significance tests based on DSC values, with a p-value below 0.05 indicating statistical significance. GaussianDIR achieved a higher DSC of 81.3±2.3% and a lower HD95 of 1.89±0.50mm on the OASIS dataset compared to previous methods, and also attained the highest accuracy on the ACDC dataset. Statistical testing on the OASIS dataset showed that GaussianDIR provides significant improvements over all competing methods. Additional performance comparisons and significance analyses are provided in Supplementary Material Section B.

**Table 1:** Quantitative results, including mean (standard deviation) DSC, HD95 and NJD, on the OASIS dataset and the ACDC dataset. The best results are bold. ‘Classical’ denotes conventional methods, ‘DL’ means deep-learning-based methods, while ‘INR’ represents implicit neural representation.

Models	Type	OASIS			ACDC		
		DSC (%) ↑	HD95 (mm) ↓	NJD (%) ↓	DSC (%) ↑	HD95 (mm) ↓	NJD (%) ↓
NiftyReg	Classical	77.6 (3.1)⁎	2.33 (0.76)	7.596 (3.14)	68.7 (13.6)	4.64 (1.91)	0.967 (2.75)
SyN	Classical	78.7 (3.1)⁎	2.16 (0.55)	0.008 (0.01)	73.8 (9.3)	4.13 (1.52)	0.022 (0.09)
FireANTs	Classical	80.5 (2.9)⁎	1.96 (0.49)	**0**	79.5 (6.3)	3.47 (1.30)	0.003 (0.01)
Greedy	Classical	80.6 (2.7)⁎	1.94 (0.51)	**0**	80.0 (6.1)	3.47 (1.31)	**0.002 (0.01)**
Demons	Classical	80.2 (2.4)⁎	2.02 (0.46)	0.094 (0.03)	79.4 (6.2)	3.47 (1.28)	3.400 (1.98)
SymNet	DL	74.8 (–)⁎	–	–	–	–	–
LapIRN	DL	78.8 (–)⁎	–	–	79.0 (–)	–	0.454 (–)
CorrMLP	DL	–	–	–	81.0 (–)	–	0.389 (–)
NODEO	INR	79.0 (2.7)⁎	2.05 (0.48)	0.013 (0.01)	80.3 (5.7)	**3.33 (1.28)**	0.008 (0.03)
IDIR	INR	78.1 (2.6)⁎	2.25 (0.62)	1.191 (0.21)	80.6 (5.3)	3.55 (1.33)	0.496 (0.38)
ccIDIR	INR	78.4 (2.6)⁎	2.16 (0.58)	0.763 (0.13)	80.0 (5.7)	3.65 (1.47)	0.113 (0.15)

GaussianDIR	Gaussian	**81.3 (2.3)**	**1.89 (0.50)**	1.091 (0.24)	**81.2 (5.3)**	3.43 (1.26)	0.347 (0.32)

* P<0.05, in comparison to GaussianDIR.

### 3.2 Generalization

As shown in Table 2, although Transmorph achieved high accuracy with weak supervision, outperforming GaussianDIR on OASIS dataset, its performance declined extremely when applied to the unseen IXI dataset, another brain MRI dataset without an obvious domain gap as OASIS.

**Table 2:** Generalization comparisons between DL-based weak-supervised TransMorph and GaussianDIR.

Datasets	Methods	DSC (%) ↑	HD95 (mm) ↓	NJD (%) ↓
OASIS	TransMorph	85.7 (1.4)	1.45 (0.33)	0.87 (0.21)
OASIS	GaussianDIR	81.3 (2.3)	1.89 (0.50)	1.09 (0.24)

IXI	TransMorph	69.3 (3.9)	3.79 (0.69)	0.91 (0.09)
IXI	GaussianDIR	75.6 (2.3)	2.95 (0.58)	1.25 (0.16)

## 4 Discussion

Unlike recent advances that predominantly focused on network structures, this work introduced GaussianDIR, a novel case-specific optimization method for DIR. Evaluated on brain, lung, and cardiac imaging, it demonstrated competitive or superior performance compared to classical, DL-based, and INR-based approaches, while challenging the preconception that iterative methods are inherently slow.

Most optimization-based DIR methods [8], [10], [19], [20], [21] rely on gradient descent algorithm. It has been proven that, under gradient descent, the gradient direction of the DVF is parallel to the spatial gradient of the moving image at corresponding positions, regardless of the fixed image or similarity function [37]. However, the partial derivatives of the DVF with respect to different parameterizations vary, meaning that different representations impose distinct constraints and regularization effects on the DVF, ultimately influencing registration accuracy. This study introduced a novel DIR representation based on Gaussian primitives. Their dynamic and adaptive nature enables localized regularization effects that adjust to the complexity of anatomical deformations, allowing GaussianDIR to achieve improved contour and landmark alignment.

Data-driven and optimization-based approaches are two prevailing paradigms in the field of DIR, with the question of which offers the superior solution remaining a topic of ongoing debate [18]. Data-driven approaches offer real-time inference but require extensive datasets and face challenges in generalization, while optimization-based approaches are often thought to suffer from slower convergence. Moreover, the empirical evaluations by Jena et al. [18] highlighted two key findings: first, optimization-based methods generally achieved superior performance compared to data-driven methods in the absence of annotations. Second, although data-driven methods surpassed optimization-based methods when label maps were available (such as on the OASIS dataset), this performance advantage did not transfer to other datasets, even in comparison to their own unsupervised variants. However, both computational speed and generalization are critical for accurate dose delivery, especially in image-guided radiotherapy. A notable advantage of GaussianDIR is its enhanced computational efficiency, marking a significant breakthrough in the speed of optimization-based methods. Beyond efficiency, GaussianDIR demonstrated remarkable robustness and generalization capability in different imaging modalities and anatomical regions, as evidenced by our results on MRI and CT datasets, including brain, lung, and cardiac images.

**Fig. 4:**
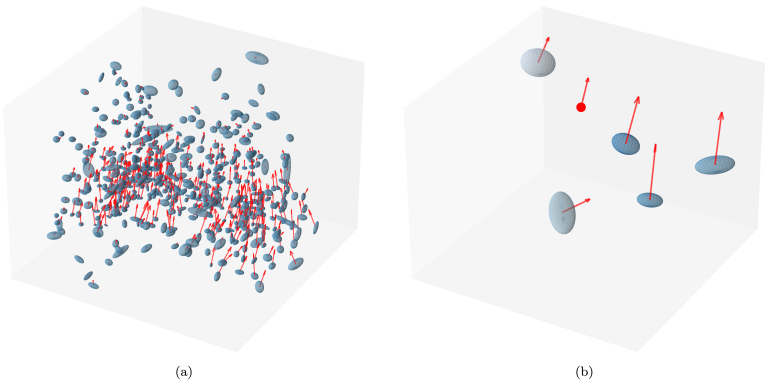
Visualization of optimized Gaussian primitives. Blue ellipses and red spheres represent the Gaussian primitives and voxel, respectively. The red arrows denote the displacement vectors of their centre positions. (For interpretation of the references to colour in this figure legend, the reader is referred to the web version of this article.)

Another key advantage of GaussianDIR is its interpretability, distinguishing it from black-box models like DL-based or INR-based approaches. By utilizing explicit Gaussian primitives, GaussianDIR makes the registration process transparent and interpretable for clinicians. Fig. 4(a) demonstrated the Gaussian primitives to aid clinical practitioners in understanding and verifying the deformation fields generated by our model. In Fig. 4(b), the ellipses are colour-coded to represent blending weights, while the red arrows denote the translation vectors of the Gaussian primitives. These visualizations allow us to evaluate whether the model produces reasonable Gaussian primitives and whether the displacements result from proper blending. Such transparency is crucial in medical settings, where trust in automated methods depends on clear, verifiable results, thus enhancing confidence in its application for clinical diagnosis and decision-making.

However, despite these promising results, there remain opportunities for further exploration. One of the current limitations is the KNN algorithm, which serves as a bottleneck in overall computational speed. Future research could explore organizing the Gaussian primitives using tree structures or implementing tile-based deformation blending with the CUDA programming language, similar to 3DGS and occupancy prediction [38], to further enhance efficiency. Another exciting avenue for future research is cross-modality registration. While GaussianDIR has demonstrated excellent performance on both MRI and CT datasets, applying it to cross-modality registration, such as aligning MRI with CT, could unlock valuable insights for radiotherapy applications. In addition, applying GaussianDIR to registration with large deformation would be promising, as its flexible and localized representation has the potential to perform better on large deformations than fixed or global representations, such as B-spline and INR. However, further exploration on setting suitable hyper-parameters for large deformation registration might be needed.

In conclusion, this study presented an optimization-based DIR approach that employed an explicit Gaussian representation to achieve efficient DVF estimation, strong generalization, and high interpretability. Future work can focus on integrating advanced data structures to accelerate KNN search and extending the framework to handle cross-modality and large-deformation registration scenarios.

## CRediT authorship contribution statement

**Jihe Li:** Methodology, Investigation, Writing - Original Draft, Visualization, Validation, Writing – review & editing. **Xiang Liu:** Validation, Writing – original draft preparation, Writing – review & editing, Resources. **Fabian Zhang:** Writing – original draft, Writing – review & editing. **Xixin Cao:** Writing – review & editing. **Joachim M. Buhmann:** Writing – review & editing. **Ye Zhang:** Writing – review & editing. **Xia Li:** Conceptualization, Methodology, Validation, Supervision, Writing – review & editing.

## Declaration of competing interest

The authors declare that they have no known competing financial interests or personal relationships that could have appeared to influence the work reported in this paper.

## Appendix A Supplementary data

MMC S1 This supplement offers a deep dive into our method, with detailed results, in-depth ablation studies, and visualizations.

## Footnotes

2 https://brain-development.org/ixi-dataset/.

## References

[1] Smolders A., Lomax A., Weber D.C., Albertini F. (2023). Patient-specific neural networks for contour propagation in online adaptive radiotherapy. Phys Med Biol.

[2] Willigenburg T., Zachiu C., Lagendijk J.J., de Boer H.C., Raaymakers B.W. (2022). Fast and accurate deformable contour propagation for intra-fraction adaptive magnetic resonance-guided prostate radiotherapy. Phys Imaging. Radiat Oncol.

[3] Nenoff L., Ribeiro C.O., Matter M., Hafner L., Josipovic M., Langendijk J.A., Persson G.F., Walser M., Weber D.C., Lomax A.J. (2020). Deformable image registration uncertainty for inter-fractional dose accumulation of lung cancer proton therapy. Radiother Oncol.

[4] Amstutz F., Nenoff L., Albertini F., Ribeiro C.O., Knopf A.C., Unkelbach J., Weber D.C., Lomax A.J., Zhang Y. (2021). An approach for estimating dosimetric uncertainties in deformable dose accumulation in pencil beam scanning proton therapy for lung cancer. Phys Med Biol.

[5] Zhang Y., Yang J., Zhang L., Court L.E., Balter P.A., Dong L. (2013). Modeling respiratory motion for reducing motion artifacts in 4D CT images. Med Phys.

[6] Fransson S., Tilly D., Ahnesjö A., Nyholm T., Strand R. (2021). Intrafractional motion models based on principal components in magnetic resonance guided prostate radiotherapy. Phys Imaging. Radiat Oncol.

[7] Thirion J.-P. (1998). Image matching as a diffusion process: an analogy with Maxwell’s demons. Med Image Anal.

[8] Beg M.F., Miller M.I., Trouvé A., Younes L. (2005). Computing large deformation metric mappings via geodesic flows of diffeomorphisms. Int J Comput Vis.

[9] Vishnevskiy V., Gass T., Szekely G., Tanner C., Goksel O. (2016). Isotropic total variation regularization of displacements in parametric image registration. IEEE Trans Med Imaging.

[10] Siebert H., Hansen L., Heinrich M.P. (2021). Int. conf. med. image comput. comput. assist. interv..

[11] Jena R., Chaudhari P., Gee J.C. (2024).

[12] Castillo R., Castillo E., Guerra R., Johnson V.E., McPhail T., Garg A.K., Guerrero T. (2009). A framework for evaluation of deformable image registration spatial accuracy using large landmark point sets. Phys Med Biol.

[13] Balakrishnan G., Zhao A., Sabuncu M.R., Guttag J., Dalca A.V. (2019). Voxelmorph: a learning framework for deformable medical image registration. IEEE Trans Med Imaging.

[14] Hoopes A., Hoffmann M., Fischl B., Guttag J., Dalca A.V. (2021). Inform. process. med. imaging.

[15] Hoffmann M., Billot B., Greve D.N., Iglesias J.E., Fischl B., Dalca A.V. (2021). SynthMorph: learning contrast-invariant registration without acquired images. IEEE Trans Med Imaging.

[16] Chen J., Frey E.C., He Y., Segars W.P., Li Y., Du Y. (2022). Transmorph: Transformer for unsupervised medical image registration. Med Image Anal.

[17] Chen Z., Zheng Y., Gee J.C. (2023). Transmatch: A transformer-based multilevel dual-stream feature matching network for unsupervised deformable image registration. IEEE Trans Med Imaging.

[18] Jena R., Sethi D., Chaudhari P., Gee J. (2024). Deep learning in medical image registration: Magic or mirage?. Adv Neural Inf Process Syst.

[19] Wolterink J.M., Zwienenberg J.C., Brune C. (2022). Int. conf. med. imaging deep learn..

[20] Van Harten L.D., Stoker J., Išgum I. (2023). Robust deformable image registration using cycle-consistent implicit representations. IEEE Trans Med Imaging.

[21] Li X., Li M., Lomax A., Buhmann J., Zhang Y. (2024).

[22] Kerbl B., Kopanas G., Leimkühler T., Drettakis G. (2023). 3D Gaussian splatting for real-time radiance field rendering. ACM Trans Graph.

[23] Modat M., Ridgway G.R., Taylor Z.A., Lehmann M., Barnes J., Hawkes D.J., Fox N.C., Ourselin S. (2010). Fast free-form deformation using graphics processing units. Comput Methods Programs Biomed.

[24] Vercauteren T., Pennec X., Perchant A., Ayache N. (2009). Diffeomorphic demons: Efficient non-parametric image registration. NeuroImage.

[25] Rao Y.R., Prathapani N., Nagabhooshanam E. (2014). Application of normalized cross correlation to image registration. Int J Res Eng Technol.

[26] Marcus D.S., Wang T.H., Parker J., Csernansky J.G., Morris J.C., Buckner R.L. (2007). Open access series of imaging studies (OASIS): cross-sectional MRI data in young, middle aged, nondemented, and demented older adults. J Cogn Neurosci.

[27] Bernard O., Lalande A., Zotti C., Cervenansky F., Yang X., Heng P.-A., Cetin I., Lekadir K., Camara O., Ballester M.A.G. (2018). Deep learning techniques for automatic MRI cardiac multi-structures segmentation and diagnosis: is the problem solved?. IEEE Trans Med Imaging.

[28] Meng M., Feng D., Bi L., Kim J. (2024). IEEE conf. comput. vis. pattern recog..

[29] Zhou S., Hu B., Xiong Z., Wu F. (2023). Self-distilled hierarchical network for unsupervised deformable image registration. IEEE Trans Med Imaging.

[30] Sharp G.C., Li R., Wolfgang J., Chen G., Peroni M., Spadea M.F., Mori S., Zhang J., Shackleford J., Kandasamy N. (2010). ICCR.

[31] Rühaak J., Polzin T., Heldmann S., Simpson I.J., Handels H., Modersitzki J., Heinrich M.P. (2017). Estimation of large motion in lung CT by integrating regularized keypoint correspondences into dense deformable registration. IEEE Trans Med Imaging.

[32] Mok T.C., Chung A. (2020). IEEE conf. comput. vis. pattern recog..

[33] Mok T.C., Chung A.C. (2020). Int. conf. med. image comput. comput. assist. interv..

[34] Jiang Z., Yin F.-F., Ge Y., Ren L. (2020). A multi-scale framework with unsupervised joint training of convolutional neural networks for pulmonary deformable image registration. Phys Med Biol.

[35] Hering A., Häger S., Moltz J., Lessmann N., Heldmann S., Van Ginneken B. (2021). CNN-based lung CT registration with multiple anatomical constraints. Med Image Anal.

[36] Wu Y., Jiahao T.Z., Wang J., Yushkevich P.A., Hsieh M.A., Gee J.C. (2022). IEEE conf. comput. vis. pattern recog..

[37] Jena R., Chaudhari P., Gee J.C. (2025). Deep implicit optimization enables robust learnable features for deformable image registration. Med Image Anal.

[38] Huang Y., Zheng W., Zhang Y., Zhou J., Lu J. (2024). Eur. conf. comput. vis..

