# Supplementary material for: Gaussian primitives for deformable image registration
Source: Phys Imaging Radiat Oncol. 2025 Aug 8;35:100821. doi: 10.1016/j.phro.2025.100821 (PMC12361785; doi:10.1016/j.phro.2025.100821)
Supplement: MMC S1 — This supplement offers a deep dive into our method, with detailed results, in-depth ablation studies, and visualizations. [file mmc1.pdf]

Supplementary Material: Gaussian Primitives for Deformable Image Registration

A. ADDITIONAL METHOD EXPLANATION

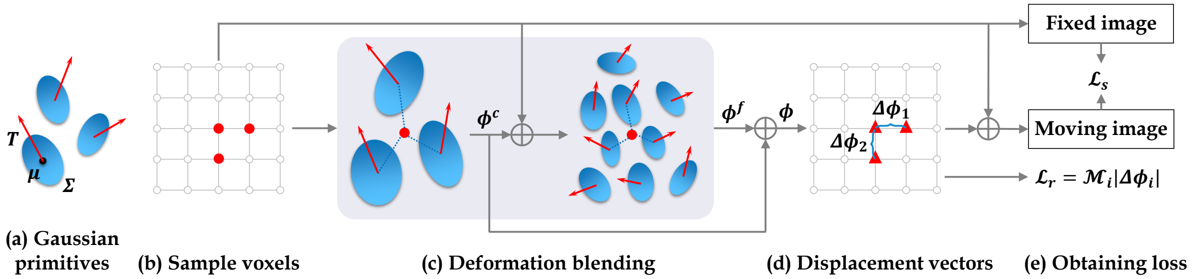

Fig. S1. Pipeline of GaussianDIR. In contrast to Figure 1 in main paper, this figure presents a more detailed depiction of the multiscale Gaussian primitives. (a) Blue ellipses represent 3D Gaussians parameterized by centre position $\boldsymbol{\mu}$ and covariance matrix $\boldsymbol{\Sigma}$. Red arrows indicate the local deformations $\boldsymbol{T}$. (b) Voxel groups are sampled from the volume to enable mini-batch optimization and total variation regularization. (c) Local deformations of neighbouring Gaussian primitives are blended, with neighbours identified using the K-nearest neighbour (KNN) algorithm at two different scales. (d) Deformation vectors $\boldsymbol{\phi}$ are obtained after deformation blending. (e) The similarity loss $\mathcal{L}_s$ and regularization loss $\mathcal{L}_r$ are then calculated. $\mathcal{M}$ represents the mean operation.

A.1. Adaptive Density

The density of Gaussian primitives is a critical hyperparameter, directly influencing the complexity of the DVF modelling. Striking a delicate balance is critical: excessively dense Gaussian primitives can lead to over-fitting to noise, thereby compromising the smoothness of the resultant deformation field. Conversely, too sparse Gaussian primitives may result in underfitting, particularly in regions with complex deformations. Manually adjusting this hyperparameter for each pair of images is both inefficient and impractical.

To address this challenge, we employ an adaptive density control scheme inspired by previous work [1], where the density of Gaussian primitives is dynamically adjusted. Specifically, we determine whether a Gaussian primitive should be cloned or pruned based on the norm of its position gradient norm. Gaussian primitives with gradient norm exceeding a predefined threshold $\boldsymbol{\tau}_{\max} = 0.002$ are cloned, while those with norm below $\boldsymbol{\tau}_{\min} = 1 \times 10^{-7}$ are pruned. The cloning process involves duplicating a Gaussian primitive $\boldsymbol{G}_i$ by sampling a random displacement $\Delta\boldsymbol{\mu}_i$ from a 3D normal distribution $\boldsymbol{N}(\boldsymbol{0}, \boldsymbol{s}_i)$, where $\boldsymbol{s}_i$ denotes the scaling vector of $\boldsymbol{G}_i$. The coordinate $\hat{\boldsymbol{\mu}}_i$ of the new Gaussian is determined by adding this random displacement to the original position as $\hat{\boldsymbol{\mu}}_i = \boldsymbol{\mu}_i + \Delta\boldsymbol{\mu}_i$, while the other parameters are retained. The gradient of new Gaussian primitives is initially set as zeros and will be calculated through back-propagation in the next iteration. Pruning is achieved by straightforwardly removing the corresponding Gaussian primitives. These operations are conducted every $0.05 \times M$ iterations.

A.2. Multi-scale Gaussian Primitives

The multi-scale scheme is commonly employed to mitigate the impact of local minima, either through image pyramids [2] or pyramid networks [3]. In our framework, we progressively refine the DVF across multiple-scale Gaussian primitives without downsampling the original images. This is achieved by initializing Gaussian primitives at multiple levels of granularity, with varying maximum numbers of primitives across different scales. The number of Gaussian primitives directly influences the complexity of the deformation field, allowing for a coarse-to-fine refinement of the DVF. As illustrated in Figure S1(c), we begin by estimating a coarse deformation field $\boldsymbol{\phi}^c$ using larger, sparser Gaussian primitives. This is followed by a finer deformation field $\boldsymbol{\phi}^f$ derived from smaller, denser Gaussian primitives. The final DVF $\boldsymbol{\phi}$ is obtained by combining these fields: $\boldsymbol{\phi} = \boldsymbol{\phi}^c + \boldsymbol{\phi}^f$. This multi-scale facilitates reducing computational complexity while maintaining precision. During optimization process, the optimization is divided into two stages. In the first stage, we set the maximum number of Gaussian primitives to $\frac{1}{8} \times |\Omega|$, while in the second stage, the number is increased to $\frac{1}{4} \times |\Omega|$, starting at the $0.5 \times M$ iteration.

A.3. Loss Function

The loss function consists of a similarity term $\mathcal{L}_s$ and a regularization term $\mathcal{L}_r$. As described in the main paper, we employ negative normalized cross-correlation (NCC) [4] as the similarity loss and a mini-batch-based total variation (TV) loss as the regularization. The similarity loss is defined as:

$$\mathcal{L}_s(I_f, I_w) = -\frac{\sum_{x_i}(I_f(x_i) - \bar{I}_f)(I_w(x_i) - \bar{I}_w)}{\sqrt{\sum_{x_i}(I_f(x_i) - \bar{I}_f)^2 \sum_{x_i}(I_w(x_i) - \bar{I}_w)^2}}, \tag{S1}$$

where $\bar{I}_f$ and $\bar{I}_w$ denote the mean intensities of I_f and I_w, and x_i indexes voxel locations, all within each mini-batch. The mini-batch-based TV loss is given by:

$$\mathcal{L}_r(\phi) = \frac{1}{B}\sum_{j=0}^{B-1}\sum_{d=1}^{D}||\phi_{jd} - \phi_j||_2, \tag{S2}$$

where ϕ_{jd} denotes the displacement vector at the neighboring voxel of x_j along orthogonal direction d.

B. ADDITIONAL EXPERIMENTAL RESULTS

B.1. Performance Comparisons

Detailed case-wise TRE comparisons on the DIR-Lab dataset are presented in Table S1. Notably, GaussianDIR surpasses most methods in TRE, with the exception of pTV [2]. However, our approach is about 16 times faster than pTV. The statistical significance test on DIRLab dataset shows that GaussianDIR presents statistically significant improvements over all competing methods on DIRLab dataset, except for pTV [2] (p = 0.57), demonstrating that GaussianDIR significantly outperforms most methods while showing no substantial registration degradation compared to pTV. Figure S2 shows error maps for Case 8 from DIRLab after approximately 2.5 seconds of optimization. The depth of the color indicates the magnitude of the error, with deeper colors representing larger errors. Compared to other methods, GaussianDIR exhibits substantially smaller errors under equivalent runtime. Figure S3 illustrates comparisons of warped moving images and the corresponding DVFs. The highlighted regions within the red boxes indicate that the warped moving image generated by GaussianDIR aligns more closely with the fixed images. Furthermore, the DVFs highlight GaussianDIR's strong edge-preserving capabilities, which are crucial for maintaining anatomical consistency. Figure S4 shows error maps from the ACDC dataset, indicating that GaussianDIR achieves lower registration errors than ccIDIR and IDIR within the same optimization time of approximately 1.2 seconds.

Table S1. Evaluation of TRE (↓) in *mm* on the DIR-Lab dataset. Results for GaussianDIR are averaged over 5 random seeds. Time is represented in seconds. *: $P < 0.05$, in comparison to GaussianDIR. VM denotes VoxelMorph.

Methods	GaussianDIR	ccIDIR*	IDIR*	pTV	ConvexAdam*	Demons*	FireANTs*	VIRNet*	MJCNN*	VM*
Time	5.1	50.3	20.2	80.1	1.6	138.1	795.4	0.8	1.4	0.8
CT 01	0.78 (0.92)	0.83 (0.94)	0.76 (0.94)	0.76 (0.90)	1.00 (1.03)	1.07 (0.50)	0.92 (0.96)	0.99 (0.47)	1.20 (0.63)	1.03 (1.01)
CT 02	0.75 (0.90)	0.78 (0.93)	0.76 (0.94)	0.77 (0.89)	0.74 (0.96)	1.14 (0.97)	0.89 (0.96)	0.98 (0.46)	1.13 (0.56)	1.09 (1.87)
CT 03	0.94 (1.06)	1.02 (1.10)	0.94 (1.02)	0.90 (1.05)	1.02 (1.11)	1.32 (0.80)	1.09 (1.08)	1.11 (0.61)	1.30 (0.70)	1.40 (2.04)
CT 04	1.25 (1.24)	1.37 (1.36)	1.32 (1.27)	1.24 (1.29)	1.39 (1.35)	1.86 (1.67)	1.54 (1.47)	1.37 (1.03)	1.55 (0.96)	1.69 (2.60)
CT 05	1.11 (1.46)	1.25 (1.51)	1.23 (1.47)	1.12 (1.44)	1.42 (1.63)	1.75 (1.65)	1.42 (1.57)	1.32 (1.36)	1.72 (1.28)	1.63 (2.44)
CT 06	0.96 (1.00)	1.06 (1.09)	1.09 (1.03)	0.85 (0.89)	1.31 (1.25)	2.33 (2.82)	2.24 (3.14)	1.15 (1.12)	2.02 (1.70)	1.60 (2.58)
CT 07	0.93 (0.96)	0.97 (0.98)	1.12 (1.00)	0.80 (1.28)	1.49 (1.73)	4.18 (4.84)	3.68 (5.57)	1.05 (0.81)	1.70 (1.03)	1.93 (2.80)
CT 08	1.10 (1.26)	1.13 (1.40)	1.21 (1.29)	1.34 (1.93)	2.91 (5.13)	6.92 (8.77)	7.17 (9.50)	1.22 (1.44)	2.64 (2.78)	3.16 (4.69)
CT 09	1.00 (0.95)	1.02 (0.93)	1.22 (0.95)	0.92 (0.94)	1.33 (1.32)	1.56 (1.18)	1.57 (1.92)	1.11 (0.66)	1.51 (0.94)	1.95 (2.37)
CT 10	0.92 (0.90)	0.96 (0.98)	1.01 (1.05)	0.82 (0.89)	1.41 (2.11)	2.43 (3.24)	1.94 (3.40)	1.05 (0.72)	1.79 (1.61)	1.66 (2.87)
Average	0.97 (1.07)	1.04 (1.12)	1.07 (1.10)	0.95 (1.15)	1.40 (1.76)	2.45 (1.72)	2.24 (1.82)	1.14 (0.76)	1.66 (1.44)	1.71 (2.86)

B.2. Ablation Studies

This section evaluates the impact of various design choices in our proposed GaussianDIR, shedding light on their individual and collective contributions to the overall performance and robustness of the approach. All the ablation experiments are conducted on the OASIS dataset with 49 pairs except the generalization comparison in Section B.2.

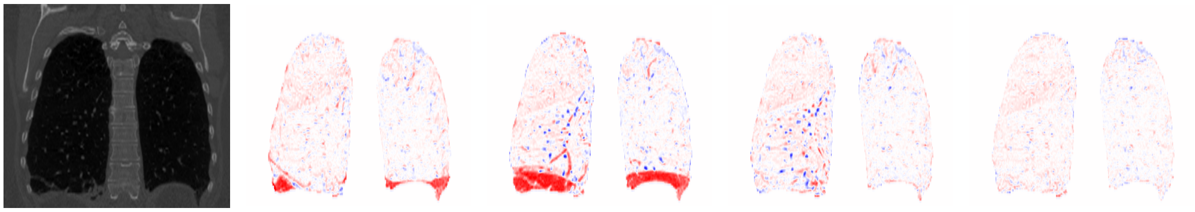

(a) Fixed Image **(b)** ConvexAdam [5] **(c)** ccIDIR [6] **(d)** IDIR [7] **(e)** GaussianDIR

Fig. S2. Error maps for Case 8 of DIRLab dataset. This comparison shows error maps generated by four methods, each trained for approximately 2.5 seconds. The color's depth indicates the error's magnitude, with deeper colors representing larger errors.

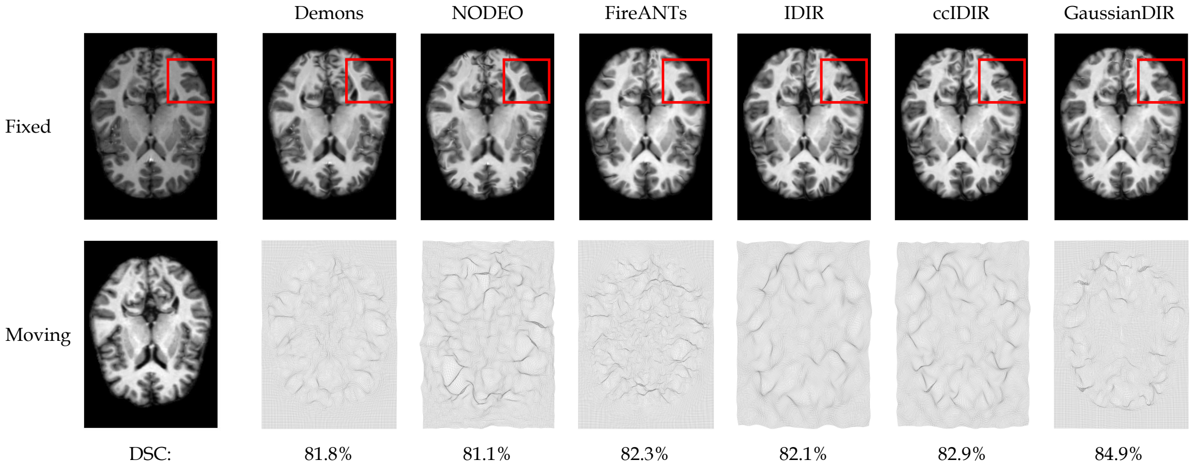

Fig. S3. Visualization of warped moving images and deformation fields on the OASIS dataset. Their corresponding DSC values are presented in the bottom row. The highlighted regions within the red boxes indicate that the warped moving image from GaussianDIR aligns more closely with the fixed images.

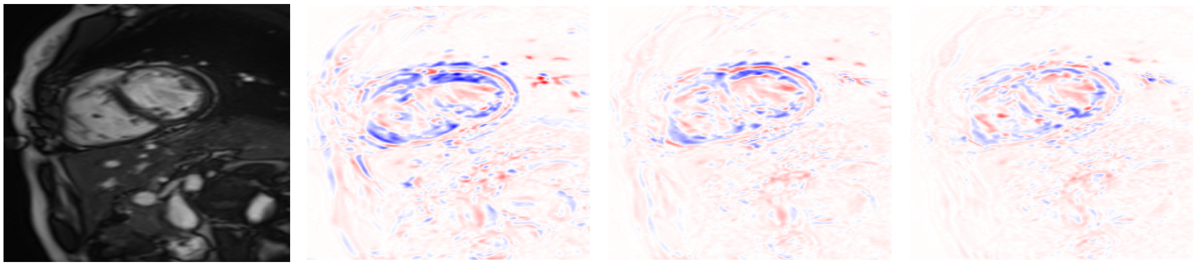

(a) Fixed Image **(b)** ccIDIR [6] **(c)** IDIR [7] **(d)** GaussianDIR

Fig. S4. Visualization of error maps for different methods trained for approximately 1.2 seconds. The depth of color indicates the magnitude of the error, with deeper colors representing larger errors.

Free Gaussian GaussianDIR leverages fully flexible Gaussian primitives whose positions and shapes are capable of adapting to tissue deformation and structure. We evaluate the impact of using free versus fixed Gaussian primitives on registration accuracy. As shown in Table S2, allowing for free Gaussian primitives yields a 1.4% improvement in DSC and a reduction of 0.06 in HD95.

Anisotropic Gaussian and 6-DoF Rigid Deformation Isotropic Gaussians, constrained to spherical shapes, are less effective in capturing the nuances of tissue structures. In contrast, anisotropic Gaussians offer a more flexible representation. Table S2 shows that employing anisotropic Gaussian primitives improves the DSC from 81.0% to 81.2% and reduces HD95 from 1.92 to 1.91. Moreover, introducing a 6-DoF rigid deformation, which combines both rotation and translation, further enhances performance.

Robustness on Initialization We assess the robustness of GaussianDIR to different initialization strategies by comparing random and grid-based initialization. Our experiments reveal that GaussianDIR achieves consistent performance across both methods. A statistical significance test yields a p-value of 0.68, indicating no statistically significant difference in results between the two strategies at the 5% significance level.

Table S2. Quantitative evaluation for ablation runs. Aniso., Rot. and Init. denote employing anisotropic Gaussian, 6-DoF rigid deformation rather than translation only, and initialization strategy, respectively.

Gaussian	Aniso.	Rot.	Init.	DSC (%) ↑	HD95 ↓	NJD (%) ↓
Fixed	✗	✗	Grid	79.6 (2.5)	1.98 (0.51)	0.87 (0.23)
Free	✗	✗	Grid	81.0 (2.3)	1.92 (0.50)	1.16 (0.24)
Free	✓	✗	Grid	81.2 (2.3)	1.91 (0.51)	1.22 (0.25)
Free	✓	✓	Grid	81.3 (2.3)	1.89 (0.50)	1.09 (0.24)
Free	✓	✓	Random	81.3 (2.3)	1.89 (0.52)	1.09 (0.24)

Numbers of Gaussian Primitives and Adaptive Density We conducted a series of experiments to evaluate how target registration error (TRE) varies with the number of Gaussian primitives and to compare performance with and without the adaptive density scheme. As shown in Figure S5, GaussianDIR demonstrates robustness across a range of primitive counts; however, an excessive or insufficient number of primitives leads to degraded performance. Notably, GaussianDIR without adaptive density consistently underperforms its adaptive counterpart. We attribute this to underfitting in cases with too few primitives and overfitting to noise when too many are used. In contrast, the adaptive density scheme adjusts the primitive number to match deformation complexity, achieving a better trade-off between capturing meaningful signal and suppressing noise.

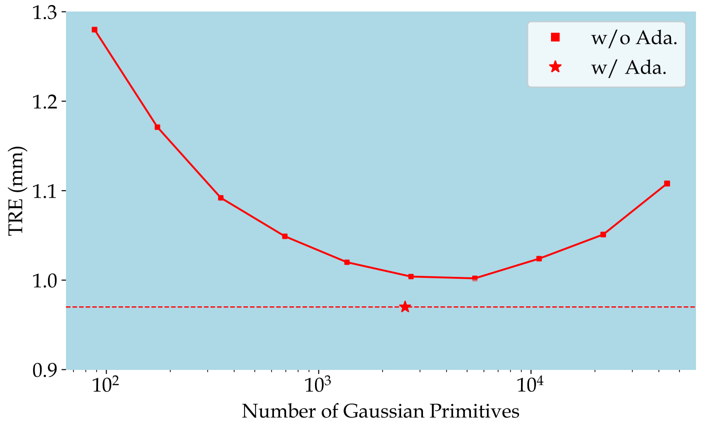

Fig. S5. Variation of TRE with respect to the number of Gaussian primitives, and comparison between GaussianDIR with (w/) and without (w/o) adaptive density (Ada.).

REFERENCES

1. Kerbl B, Kopanas G, Leimkühler T, Drettakis G. 3d gaussian splatting for real-time radiance field rendering. ACM Trans Graph 2023;42(4):1–14. doi:https://doi.org/10.1145/3592433.
2. Vishnevskiy V, Gass T, Szekely G, Tanner C, Goksel O. Isotropic total variation regularization of displacements in parametric image registration. IEEE Trans Med Imaging 2016;36(2):385–395. doi:https://doi.org/10.1109/TMI.2016.2610583.
3. Zhou S, Hu B, Xiong Z, Wu F. Self-distilled hierarchical network for unsupervised deformable image registration. IEEE Trans Med Imaging 2023;42(8):2162–2175. doi:https://doi.org/10.1109/TMI.2023.3244333.
4. Rao YR, Prathapani N, Nagabhooshanam E. Application of normalized cross correlation to image registration. Int J Res Eng Technol 2014;3(5):12–16. doi:https://doi.org/10.15623/ijret.2014.0317003.
5. Siebert H, Hansen L, Heinrich MP. Fast 3d registration with accurate optimisation and little learning for learn2reg 2021. In: Int Conf Med Image Comput Comput Assist Interv. Springer; 2021, p. 174–179. doi:https://doi.org/10.1007/978-3-030-97281-3_25.
6. Van Harten LD, Stoker J, Išgum I. Robust deformable image registration using cycle-consistent implicit representations. IEEE Trans Med Imaging 2023;43(2):784–793. doi:https://doi.org/10.1109/TMI.2023.3321425.
7. Wolterink JM, Zwienenberg JC, Brune C. Implicit neural representations for deformable image registration. In: Int Conf Med Imaging Deep Learn. PMLR; 2022, p. 1349–1359.
